# Ten Years of Experience in Contraception Options for Teenagers in a Family Planning Center in Thrace and Review of the Literature

**DOI:** 10.3390/ijerph15020348

**Published:** 2018-02-15

**Authors:** Panagiotis Tsikouras, Dorelia Deuteraiou, Anastasia Bothou, Xanthi Anthoulaki, Anna Chalkidou, Eleftherios Chatzimichael, Fotini Gaitatzi, Bachar Manav, Zacharoula Koukouli, Stefanos Zervoudis, Grigorios Trypsianis, George Galazios

**Affiliations:** 1 Department of Obstetrics and Gynecology, Democritus University of Thrace, Alexandroupolis 68100, Greece; Dr.Dorelia@hotmail.com (D.D.); xanthi_vatsidou@hotmail.com (X.A.); annachalkidou@yahoo.gr (A.C.); elevchat7@med.duth.gr (E.C.); pan.bougatsou@gmail.com (F.G.); bacharmanav@windowslive.com (B.M.); harakoukouli@gmail.com (Z.K.); ggalaz@med.duth.gr (G.G.); 2 Department of Obstetrics and Mastology, Rea Hospital, Athens 17564, Greece; natashabothou@windowslive.com (A.B.); szervoud@otenet.gr (S.Z.); 3 Department of Medical Statistic, Democritus University of Thrace, Alexandroupolis 68100, Greece; gtryps@med.duth.gr

**Keywords:** adolescents, contraception’s options, health counseling center, family planning center

## Abstract

*Introduction*: The goal of our study was to investigate and evaluate the contraceptive behavior in teenagers from our family planning centre that services two different religious and socioeconomic populations living in the Thrace area. *Methods*: During the last 10 years 115 Christian Orthodox (group A) and 53 Muslim teenagers (group B) were enrolled in our retrospective study. Contraceptive practice attitudes were assessed by a questionnaire. Religion, demographics, socio-economic characteristics were key factors used to discuss contraception and avoid unplanned pregnancy in each group and to compare with the contraceptive method used. *Results*: The most used contraceptive method—about two times more frequently—among Christian Orthodox participants was the oral contraceptive pill (*p* = 0.015; OR = 1.81, 95% CI = 1.13–2.90), while in the other group the use of condoms and IUDs was seven and three times more frequent, respectively. Our family planning centre was the main source of information for contraception. *Conclusions*: During adolescence, the existence of a family planning centre and participation in family planning programs plays a crucial role to help the teenagers to improve their knowledge and choose an effective contraception method.

## 1. Introduction

Reproductive health must be based on the freedom of young people to access appropriate health care services. There they can be easily informed about all the possible fertility regulation methods and make decisions for having a satisfying, safe sex life with preservation of reproductive capability [1]. The contraception choices for teenager women significantly affect their health, their school and professional education as well as their smooth transition to adulthood [2]. According to international literature, the overwhelming majority of adolescents do not want pregnancies [3]. Those who are already married also do not want childbirth and those who become pregnant do not wish the birth of another child [3]. The world population of adolescents aged 10–19 years now stands at 1.2 billion [4]. It is essential to understand and approach their sexual needs. Unfortunately, most teenagers do not have access to the counselling and services they need. More than 50% of US women have sexual contacts before the age of 18. Of these, 1/3 does not use any contraception [5].

In developing countries, most pregnancies in adolescence are unexpected and undesirable, and as a result the half of them end up with an *induced abortion* taking place, very often under unsafe conditions [6,7]. In 2011, 47% of high-school students reported having some sexual experience and 34% reported sexual contact in the last three months [8]. Annually some 750.000 teenage pregnancies are reported and 82% of these pregnancies were unplanned [9]. From these reported pregnancies observed 59% births, 14% miscarriage and 27% induced abortion. From1990 to early 2000, a significant reduction in teenage pregnancies has been observed. The majority (86%) of this decrease is due to the increased use of contraception and 14% due to abstention or delay in starting sexual contact [10]. In Greece this percentage is much smaller, but existent. Elements from the 2007 MES study in Attica are the following:

(a) Up to 16 years of age 20% of adolescents (in a 1 to 3 proportion of boys to girls) have started their sexual life,
(b) Among sexually active adolescents, 5.7%, 10.2%, 44.3%, 33% and 2.3% started sex at 12, 13, 14, 15 and 16 years of age, respectively,
(c) 40% of adolescents had some sexual experience other than vaginal sex [11].

According to the Greek data Adolescence Health Unit (AHU) Youth-health Study, among sexually active teenagers:

(a) 10% used no contraceptive method,
(b) 39% used unreliable methods, such as rhythm or withdrawal methods,
(c) 51% used condoms, and
(d) 5% the oral contraceptive pill.

Therefore, the contraception use of teenagers is a real challenge for the medical community and sometimes can be difficult, especially for those suffering from a chronic disease. This is because in several such cases, taking medication can affect the safety and effectiveness of hormonal contraception, and as a result a pregnancy with high maternal and fetal risk is unavoidable. The need for contraceptive protection of adolescents with chronic problems or special needs very often is overlooked. It is estimated that this category includes 10–20% of the population under the age of 20 [12,13,14]. For all the above reasons, the need for sexual education and knowledge of contraception choices and abilities of these people should not be overlooked [12,13,14]. Recent data showed that there are no differences in the sexual experience of these individuals. Contraception is very important factor worldwide to save and an immense amount of time, energy and money according to *prevention* of unintended pregnancies, especially in adolescents [12,13,14]. The use and mode of contraception is influenced from various determinants like demographic characteristics, education, occupation, religion, and social status [12,13,14]. The goal of this retrospective study was to investigate the contraceptive behavior of teenagers in two different ethnological and socioeconomic populations in the Thrace area.

## 2. Methods

From January 2006 until December 2016 we studied the different attitudes towards contraception, among females of two major community subgroups: 115 Christian Orthodox women living in Thrace, Greece (group A) and 53 Muslim women living in Thrace, Greece (group B).

All respondents were in reproductive adolescence age (from 15 to 19 years) and were randomly selected from among teenagers visiting the Family Planning Centre in the Department of Obstetrics and Gynecology of our Hospital, the University Hospital of Alexandroupolis. The study participants either in the Group A or Group B were healthy, without past diseases. They typically visited our centre with their parents for a routine gynaecologic examination and attitudes concerning contraceptive practices were assessed by means of a questionnaire. Each question was explained to the participants, who subsequently completed the questionnaire in private and finally gave it back during the next examination. For the teenagers with relatively late occurring menstruation, acne, BMI greater than 25 Kg/m^2^ and hirsutism hormonal laboratory and vaginal ultrasound examinations were performed to investigate possible polycystic ovary (PCO) syndrome.

All teenagers who with their parents’ consent were included in the study were very cooperative in answering the questions and gave anonymous detailed answers regarding their age, place of living, religion, occupation, economic and social status, menarche, and menstrual bleeding abnormalities status. As well as the main source of information offered to them about contraception, the above mentioned characteristics of each group were compared with the method of contraception used.

Statistical analysis of the data was performed using the Statistical Package for the Social Sciences (SPSS), version 19.0 (SPSS, Inc., Chicago, IL, USA). All variables were categorical and they were expressed as frequencies and percentages (%). The chi-square test was used to evaluate any potential association between the method of contraception and the participants’ characteristics; odds ratios (OR) and their 95% confidence intervals (95% CI) were calculated by means of simple logistic regression analysis, as the measure of the above associations. All tests were two tailed and statistical significance was considered for *p*< 0.05.

## 3. Results

The demographic characteristics of the study participants are listed in Table 1. The vast majority of the participants (267 teenagers, 89.0%) used some method of contraception; the contraceptive pill (56.0%), interrupted coitus (45.7%) and the use of condoms (42.3%) were the most common methods of contraception (Table 2). The use of two different methods of contraception was observed in 41.3% of the teenagers. While35.7% of the teenagers used only one method of contraception, and three or four methods were used by 10.0% and 2.0% of the teenagers, respectively (Table 3a). The family center (47.0%) was the most usual source of information regarding contraception; a family consultant (20.3%), school (20.3%) and the sexual partner, newspaper, news or media (12.4%) were other sources of information (Table 3b).

The association of the use of different methods of contraception according to teenagers’ characteristics is shown in Table 4 and Table 5. The use of the contraceptive pill was almost twice more frequent among Christian Orthodox (*p*=0.015; OR = 1.81, 95% CI =1.13–2.90), among teenagers living in rural areas (*p* =0.012), while a tendency towards more frequent use of the contraceptive pill was found at higher education levels (*p*= 0.077).

On the contrary, the use of condoms and IUDs were almost seven and three times more frequent among Muslims (condom: *p*< 0.001; OR = 6.73, 95% CI = 4.01–11.31; IUD: *p* = 0.007; OR = 2.71, 95% CI = 1.28–5.72), respectively. Moreover, the use of IUD and spermicide were more frequent among employed teenagers (*p* = 0.087 and *p* = 0.007, respectively). No other statistically significant associations were found.

One hundred and fourteen teenagers had clinical symptoms (late menarche, acne, hirsutism, hormone laboratory results: mean serum T levels 6915 ± 509 (0.06–0.82 ng/mL) and mean serum DHEAS of 61,957 ± 11,539 μg/dL (65.1–368)). As in the entire cohort, the use of contraceptive pill (61.8%) and the use of condom (65%) were confirmed the most common methods of contraception among the 72 Christians and the 42 Muslims with clinical symptoms, respectively (Table 6).

## 4. Discussion

Family planning services are limited by governmental programs through commercial retail sales and are trying to adopt inappropriate aspects of family planning [15]. The aim of these centers should be to provide services to all, irrespective of the country’s economic situation, providing birth control methods using the medical profession and health services to adolescents to understand contraceptive options and to act quickly by choosing the most appropriate method [16,17]. There are many methods of contraception. Everyone requires education. Contraceptive methods are divided in natural, hormonal, intrauterine, spermicide, barrier methods and male contraception. Physical methods include periodic abstinence and coitus interruptus [17,18]. Effective contraceptive behaviors are not an individual achievement; on the contrary, choosing the right method depends on the increased resources that go into research and family planning services with reversible methods of contraception [17,18]. The recommend choice criteria for the individual suitable method are as follows: use of a contraceptive (CM) method in any case, no limitation on contraceptive (CM), general use CM, received benefits (of the CM) predominate over theoretical or clinically proven risks, risks override reception benefits (CM), high risk and finally use of CM only, non-existent alternative and inappropriate method for use. Information on the benefits of applying emergency contraception irrespective of the method proposed is also essential [19,20,21].

The oral contraceptive (OC) pill is the most prevalent form of contraception among adolescents with low compliance rates and increased interruption rates [22,23].Health care professionals should recommend the use of the male condom to reduce the transmission of sexually transmitted diseases. This specific and confidential approach of counselling increases the effectiveness with regard to the use and compliance of the contraceptive methods. It is considered essential to provide information about possible side effects that may arise from the use of various contraceptive methods, because familiarisation with the above, may significantly contribute in avoiding or interrupting the contraceptive method. It is also essential the information about the benefits of implementation of emergency contraception irrespectively of the method proposed [22,23].

The contraception method with OC effectiveness include: perfect use, avoidance of possibility of gestation when the use of the method is always fixed and correct, typical use, probability of pregnancy within one year of standard use of the method.

National studies involving multiple users with varying degrees of strictness of compliance forgotten pills, prolonged stickers and expired condoms are essential for the impact of contraception [22,23,24,25].

Regarding the use of hormonal contraception there is a clear contraindication in teenagers suffering from thrombophilia, hepatic dysfunction, systemic lupus erythematosus, cyanotic heart disease, pulmonary hypertension. Adolescents receiving antipsychotic or HIV medicines should be carefully monitored and encouraged to use injectable progestagens [25,26]. Obesity is a constantly growing health problem affecting 13% of adolescents. Obesity increases pregnancy-related morbidity because it is combined with the induction of delivery of emergency caesarean section, overweight birth, diabetes mellitus and gestational hypertension [26]. All of the above complications and especially hypertension are more common in obese teens. Concerning the use of contraception in obese patients, it should be mentioned that there is contraceptive protection regardless of the BMI size (lower rates of undesirable pregnancies) [27,28].There is limited and unclear information concerning the use of oral contraceptives in obese patients. The risk of deep vein thrombosis in obese patients is doubled. However, the additional absolute risk of venous thrombosis following the use of combined low-dose contraceptives is rather limited and certainly less than the corresponding risk due to pregnancy and periodontal disease [27,28,29,30].

There are not sufficient data about the pharmacokinetics of oral steroids in obese adolescents. As the main metabolic pathway of the EE is through the cytochrome p450 enzyme system, a reduction in EE clearance is expected in obese teenagers [30,31].

Additionally, a decrease in levonogestrel clearance is observed due to the EE-induced inhibition of the activity of the above enzyme system and consequent increase in Sex Hormone Binding Globulin (SHBG) [32]. All of the above may lead to a decrease in the contraceptive efficacy of oral contraceptives [33]. Generally, obese teenagers are more likely to become pregnant than non-obese because of a larger percentage of this population does not use contraceptive protection [32]. The effectiveness of combined hormonal contraception (pill, vaginal ring, patches) in obese patients is reduced [33]. The use of combined hormonal contraception does not affect body weight [34,35,36]. The use of intrauterine slow-release levodogestrel device has been associated with a slight increase in body weight [34,35,36]. Etonogestrel implants have little or no effect [37,38,39,40,41,42]. Regarding the use of methoxyprogesterone acetate there are conflicting data about a slight weight increase in adolescence. The use of hormonal methods of contraception is not associated with weight changes. Although in general, obesity decreases the effectiveness of oral contraceptive pills, they are superior in safety in comparison with barrier methods [41,42,43].

Therefore, medical information in these cases should focus on the modest reduction in efficacy and the increased but low absolute risk of vein thrombosis. The use of an intrauterine levonogestrel delivery devices may reduce the risk of obesity-related endometrial hyperplasia [44].

Despite the prevailing view, diabetes type II is more common among women of reproductive age. In puberty, the above frequency is even greater, which increases the importance of contraceptive protection. In any case, metabolic disorders may cause vascular disease.

Diabetics have a greater risk of complications in pregnancy as well as congenital disorders which makes it almost essential to seek pregnancy after restoring to normal glucose levels [45,46]. According to a recent Cochrane review comparing between hormonal and non-contraceptive diabetic women, only three studies related to this subject [47,48,49].

One of these was the comparison of the effect on the metabolism of women with type I diabetes of the intrauterine levonogestrel release device against the intrauterine copper device. No differences in daily insulin requirements between glycatedhaemoglobin levels and fasting glucose levels were observed over one year [50].

The other two studies referred to a comparison between progesterone contraceptive tablets and estrogen/progesterone combination contraceptive tablets. These studies showed stable glucose levels during treatment with most formulations [51]. Only high doses of contraceptives can cause a mild estrogen and progestogen disorders, having a slight adverse effect on the lipid profile whereas, tablets containing only progestogen have a correspondingly slight beneficial effect [52]. Concerning the choice of a suitable contraceptive method in diabetic adolescents, it seems that the predominance is towards the use of intrauterine devices. Oral administration of tablets is relatively safe among adolescents with type I and II diabetes without retinopathy, nephropathy and hypertension [52]. Oral contraceptive tablets are relatively safe in patients with the mild, non-antibody-presenting form of systemic lupus erythematosus. In severe cases, intrauterine devices are recommended [52,53,54].

In patients with HIV, the use of intrauterine devices is considered safe. The use of hormonal contraception may not adversely affect the progress of the disease or the risk of transmission [55]. However, some antiviral medications are potential modulators of hepatic enzyme activity and can therefore significantly affect the degree of hormonal contraceptive action, it is advisable in these cases to consider about prescribing of such drugs.

Women with migraines and focal neurological symptoms should prefer intrauterine devices or barrier methods because the use of hormonal methods may increase the risk of ischemic attack. It is also considered relatively safe to use hormonal contraceptives that contain only progesterone. In the case of taking antipsychotic medications, it should first be specified what type of preparation or formulations and possible interaction with combined oral hormonal contraception. Because many of them by influencing hepatic metabolism can cause a reduction in the effectiveness of contraceptive action. If there is such an interaction then either a higher 50μgE tablet (without special encouragement) should be proposed because there is more intermittent hemorrhage [56,57]. Whether applying an additional contraceptive method (e.g., a condom) or using an endometrial levonorgestel intrauterine device and finally use of injectable methoxyprogesterone acetate. The use of implants is not recommended due to low central progesterone levels [56,57].

Combined oral contraceptive pills are the most effective hormonal contraceptive method. The main principle of administration is based on the principle of using the lowest possible dose for the shortest possible time. Combined oral contraceptive pills contain estrogen and progestogen. Depending on the dose, circulating estrogens >50 μg (high dose), estrogens ≤35 μg (low dose) and estrogen = 20 μg (ultra-light dose). The beginning of combined oral contraceptive pills start on day 1 of menstrual cycle, followed by a 7-day discontinuation, and continuous contraceptives without interruption, and their mechanism of action consists of inhibiting implantation due to endometrial perforation and ovulation in cervical mucus thickening, sperm motility disorder, normal mobility disorder, and contraceptive action is exerted mainly via the progestogens of combined oral contraceptive pills. The estrogen exerts its contraceptive action but is dose-dependent through the inhibition of gonadotropin secretion (FSH, LH) causing the uterus to change the secretory capacity and cell structure of the endometrium [58,59,60].

Ideal progestogen pills should have strong progesterone, anti-estrogen, anti-gonadotropic action and antiglucocrticoid action. Absence of androgenic action and possible antiandrogenic action. No action similar to glucocorticoids. Lack of undesirable effects such as acne, reduction of HDL, flatulence and water retention. There are several differences regarding the hormone components contained in each formulation which can be varied depending on the type, composition, quantity and number of active tablets. The monophasic formulations contain active tablets with the same constant amount and estrogen and progestogen ratio [61,62,63,35]. In contrast to multiphasic, the above ratio changes. Biphasic ones contain two combinations and three-phase contain three. Recently there are also four phase contraceptive pills with a successive reduction in the estrogen ratio and a corresponding increase in the progestogen ratio.

Regarding levonorgestrel (2nd generation), desogestrel and gestodene (3rd generation) have greater affinity for progestagen receptors, they are more effective in inhibiting ovulation and ensuring the smoothness of the cycle at lower doses.

Regarding 1st generation contraceptive pills, there are side effects such as unwanted bleeding or spotting due to the fact that the dosage does not decrease at the appropriate time. The second generation ones are more effective and with a longer half-life, but with increased androgenic action that helps in sexual desire but could lead to hirsutism, acne and dyslipidaemia [64,65,66].

3rd generation contraceptive pills maintain efficacy of progestagen while reducing their androgenic effect and in turn, smaller androgenic effect also makes estrogen more effective. This, however, entails a greater risk for thromboembolic events.

The use of contraceptive pills results in many benefits for teenagers that are not related to contraceptive protection, such as regulating often abnormal cycles or preventing or reducing dysmenorrhoea or treating acne that is also common in teens as well as symptomatic remission of premenstrual syndrome. Continuous administration over a longer period of time (three months) is continuously gaining ground without causing particular risks. On the contrary, it improves the quality of life.

Positive effects include: restoration of a normal cycle in disorders of the cycle (hypermenorrhea, reduced blood flow, metrorrhagia, oligomenorrhea), dysmenorrhea recession through action in prostaglandin synthesis and reduction of premenstrual edema, irritability, anxiety, and depression. As far as concern ovarian and endometrial cancer can reduce the risk from 30% to 80% of endometriosis, reduce dysmenorrhoea, dysparenia and through anti-endotropic activity limit or eliminate functional, ovarian cysts. They also have a beneficial effect on the fight against acne and hirsutism by reducing androgen synthesis and specific action of estrogen and antiandrogens, causing a remission of mastodynia and many times have a positive effect on the treatment of benign breast disease. Finally they cause an increase in bone density. The latest data shows no increase of risk of breast cancer [64,66,67,68,69].

As far as concerns the 4th generation contraceptive pills, so far there are two pharmaceutical forms. The four-phase form containing a combination of estradiol valerate as estrogen and dienogest as progestagen (E2V/DNG) and the monophasic form containing 17β-oestradiol as estrogen and norgesterol acetate as progestogen (17β-E2/NMG). There are two combinations of 4th generation contraceptives: E2V/DNG and 17βE2/NOMAC. They are characterized by their particular composition including natural estrogen and 4th generation progestagens that resemble the chemical structure with progesterone. They show greater tolerability and influence are more favorable to the metabolism of carbohydrates, lipids and homeostasis because of a more favorable interference of estrogen content in liver synthesis of proteins and the antiandrogenic action of progestogen. This is safe and effective. The combination of E2V/DNG is recommended for women who experience unwanted hemorrhages due to the better regulation of the menstrual cycle. 017βE2/NOMAC is recommended in women taking contraception for the first time (new-adolescents) because of better compliance with single-phase. The use of a combination of estrogen and progesterone as a contraceptive or hormone replacement therapy has made it possible to complete large epidemiological studies that have made it possible to assess the benefits and risks that may have arisen.

The great advantages are the reduced incidence of endometrial cancer attributed predominantly to the antimitotic activity of progesterone in the endometriomas well as reducing the incidence of ovarian cancer due to mainly in the anti-endotropic activity of the above combination [67,68,69,70].

Pill Progestogen Minipill, POPs: They contain only one progestagen (usually norethindrone or norgestrel) and are used daily. The mechanism of action based on thickening of cervical mucus. Their major disadvantage is that they do not inhibit ovulation. The are used between 4 and 22 h before contact and are considered inferior to combined oral contraceptive pills [71,72].

Copper intrauterine devices: They are the most common reversible method of contraception worldwide, with a frequency of use of 30–40% among women of childbearing age in China and only 1% in the USA (due to the risk of septic automatic abortion). Their main advantage is being an easy to use method where failure does not depend from the user. They act by preventing the passage of sperm through the endometrial cavity, and the process of fertilization within the fallopian tubes. They render the ovary less capable of being fertilized and reduce the likelihood of implantation in the endometrium, probably as a result of local inflammation. They consists of a polyethylene skeleton containing wire or copper yarn. Complications include increased blood loss during menstrual bleeding and excretion. Other complications include uterine perforation, pelvic inflammation, disease PID, expulsion of intrauterine device, and ectopic pregnancy.

Contraindications for use include pregnancy suspicion, undiagnosed genital hemorrhage, and active pelvic inflammation, history of tubal pregnancy, significant deformation of the endometrial cavity and the existence of a history of bacterial endocarditis. Uterine cavity abnormalities. It is NOT contraindication: HIV infection or immunosuppression. The existence of uterine cavity abnormalities it is NOT a contraindication: HIV infection or immunosuppression (also are not contraindicated). Data for women at high risk for STIs and IUD are unclear. Nulliparous teenagers vs mothers show higher IUD rate use, pain and discomfort. In general, IUDs are a well-tolerated method with high levels of satisfaction and continuity [73].

### Barrier Methods

Male condom: It is the only reversible method of contraception for men. The average rate of failure is high (12%), while among the teenagers it is even higher (18%). It has also a significant role in limiting the transmission of sexually transmitted diseases but with some limitations. They consist of latex, polyurethane or animal membranes. New forms of male condoms made from polyurethane and their polymers seem to improve their acceptability and promise more safety. They are more protective against infections transmitted through the seminal fluid (gonococcal urethritis, chlamydia, trichomonas, HIV) than against infections transmitted through skin-to-skin contact (herpes simplex virus). Some condoms are lubricated during manufacture from the outset with the spermicide substance nanoxinol-9 which is no longer recommended due to reduced protection and higher risk for HIV. The most significant errors leading to clinical failure (ten highest among teenagers) are the rupture or displacement and the failure to use it throughout the contact [74].

Spermicides: They destroy sperm and they are available in gel, foam or cream form. They are often used simultaneously with barrier methods. The active substance is nonoxynol-9 that affects the surface of the sperm. They do not provide protection against HIV. A 2/fold to 3/fold risk of urinary tract infection during concomitant use with a barrier method is observed. They may be an appropriate option for women with sparse sexual intercourse or needing coverage for one pregnancy [75].

Polycystic ovary syndrome (PCOS): One of the most common endocrine disorders of women of reproductive age and causing hyperandrogenemia and oligo or an-ovulation. These two, lead to important psychological, social and financial consequences. Since it was found that women with PCOS manifest more often metabolic syndrome than the general population, with all its effects, there has been an increase in interest in the syndrome, both in the general population and in the medical world, in recent years. According to the currently accepted criteria include hyperandrogenism, anovulation and/or polycystic ovaries as observed by ultrasonography [76,77,78,79].

Contraceptives and PCO syndrome: Intake starts on day 1 of the menstrual cycle and it is continued for 21 days, followed by 7 days cessation. The ideal contraceptive for women with polycystic ovarian syndrome should control the development of the follicular antrum and reduce the amount of androgens, antagonise the action of androgens at the peripheral level and particularly in hair follicles and sebaceous glands and as well as restore the balance between estrogen and progestagen in the endometrium, ensuring regulation of the menstrual cycle. Administration of contraceptive tablets to women with hyperandrogenic syndrome due to PCO syndrome causes reduction in the levels of androgen circulating. The basic mechanism is the induced inhibition of maturation of follicles due to suppression of pituitary gonadotropin secretion [80,81,82,83].

In women with PCO, a contraceptive containing 30 mcg of ethinylestradiol also inhibits more effectively than lower doses of EE, steroidogenesis in adrenal glands. It also stimulates the hepatic synthesis of Sex hormone-binding globulin (SHBG). Concerning the type of progestogen, chloramandinone acetate (CMA), cyproterone acetate (CPA), dinogestor and drospirenone (DRSP) are progestagens with dynamic anandrogenicall progestagens with antiandrogenic effect. Their antiandrogenic action is mainly exerted by blocking the androgen receptors of the target organs. In addition, they reduce the action of 5α-reductase on the skin which converts testosterone to its active fraction by 5α-dihydroxytestosterone. A particular increase in SHBG is observed when using the third generation contraceptive tablets containing a combination of 30 mcg of EE with dienogest or drospirenone resulting to reduction of free testosterone by 40–50%.

The production of SHBG is to a lesser extent stimulated by second generation contraceptives containing 30 mcg of EU with levonogestrel because the androgenic action of the progestogen neutralizes the estrogenic action. Exception for the above action, some second-generation contraceptives such as the 3-phase levonogestrel, biphasic containing 30–40 mcg EU and single phase with 35 mcg EU and 2 mcg CPA. There are also studies on the efficacy of oral contraceptives containing DRSP or CPA in the treatment of hypertrichosis that show a significant reduction in circulating androgen levels after a 6–12 month treatment and a significant increase in SHBG [80,81,82,83].

Moreover, a significant decrease in the concentration of total and free testosterone was observed when the administered progestogen was CMA and DRSP. In this case the androgen concentrations are affected by those of SHBG that produce strong androgen binding. A significant decrease in dehydroandrostendione has also been observed. It is likely that this decrease is attributed to the immediate induced inhibition of adrenal androgen production. Contraceptives pills are the treatment of choice for dysfunctional bleeding in PCOS syndrome [84,85,86]. Many of the patients with the syndrome address from teenage age to specialists complaining for unexpected vaginal bleeding of various intensity and duration. The use of contraceptives in these cases in addition to the recession of hyperandrogenic phenomena also normalizes the woman’s menstrual cycle. Also, disorders of the oligo- or amenorrhea type often appear in PCOS sufferers, although 15–30% the presence of certain oligo-ovulation is associated with normal cycles. In this case, contraceptive tablets also help to restore the normality of the cycle [87,88]. Additional benefits from the use of contraceptive pills in PCOs are: The recession of dysmenorrhoea associated with menorrhagia and polymenorrhea, iron deficiency anaemia as well as the reduction of the risk of hyperplasia and endometrial cancer. There is a remarkable positive correlation between PCOS and endometrial cancer, because risk factors for endometrial cancer such as chronic anovulation, obesity, insulin resistance and diabetes mellitus are often associated with PCOS.

Although there are not enough prospective studies, it is considered reasonable to conclude that PCOS patients also benefit from the prophylactic effect of contraceptives on endometrial cancer [86,87,88].

When using contraceptive pills, the potential risk of deterioration of disorders in fat metabolism or insulin resistance should not be neglected. Estrogens lead to a dose-dependent increase in insulin resistance and the progestogens modify the above effect. Since androgens are associated with disorders in fat metabolism, progestagens with androgenic effects exhibit similarly variable gravity activity. Particularly progestagens with such activity contribute to the development of excess fat. Generally, the effect of progestogens on carbohydrate metabolism depends on the degree of hyperandrogenism of the patient and the potential antiandrogenic action of the progestogen [86,87,88].

Genetically determined susceptibility of endogenous insulin as well as anthropometric differences potentially affecting the action of insulin from the natural history of the syndrome or environmental effects. However, it is obvious that obesity and excessively high nutrition tend to define a phenotype of PCOS more prone to metabolic disorders.

Finally, contraceptive tablets are considered as risk factors for venous thrombosis, especially in carriers of genetic mutations such as factor V Leiden. The risk of arterial thrombosis, of coronary arteries is also present particularly in 35 year old women or older. This risk is directly related to the dose of estrogen (decreases when the ethinyl estradiol dose is reduced) especially in women who smoke >25 cigarettes daily. Consequently, contraceptives should not be prescribed when there are cardiovascular disorders, history of venous thrombosis, liver disease, migraine depression obesity and presence of undiagnosed mass in the breast.

Administration of contraceptive pills to women with hyperandrogenosis due to PCO syndrome causes a great reduction in the levels of circulating androgens. In women with PCOS, a contraceptive containing 30 mcg of ethinylestradiol also inhibits adrenal steroidogenesis more effectively than lower doses of EE. Regarding the type of progestogen, chloromandinone acetate (CMA), cyproterone acetate (CPA), dienogest and drospirenone (DRSP) are progestagens with dynamic antiandrogenic action [86,87,88]. Contraceptive tablets are the treatment of choice for women with PCO syndrome that have menstrual disorders either in the form of amenorrhea or oligomenorrhoea or in the form of dysfunctional bleeding because they restore the balance between estrogen and progestogens in the endometrium ensuring the normal control of the genital cycle [86,87,88].

According to our study results, we found that the most frequently contraceptive method almost twice (*p* = 0.015; OR = 1.81, 95% CI = 1.13–2.90), with improved tendency in high education levels (*p* = 0.077) is the contraceptive pill among Christian Orthodox group A in the Thrace region. In the group B, the use of condom and IUD were almost seven and three times more frequent (condom: *p* < 0.001; OR = 6.73, 95% CI = 4.01–11.31; IUD: *p* = 0.007; OR = 2.71, 95% CI = 1.28–5.72), respectively and finally the use of IUD and spermicide were more frequent among employed teenagers (*p* = 0.087 and *p* = 0.007, respectively). The above mentioned findings were confirmed also in the total subgroup with PCO symptoms distributed with parts of the both main groups (Table 6). The unexpected high percent of the usage of oral contraceptive pills compared to relatively low percent of usage in Greece generally is based on the fact that the majority of the participants were students, who have adequate education level. Additionally the variety of activities that our family planning centre offers by informing adolescents about the choices of contraception help them to make the best individual choice. In our family planning center the percentage of adolescents with acne and hirsutism, who already receiving OCP as a choice of contraception and receiving them both for therapeutic reasons of PCOS, is approximately 20% from the total population with PCOS. In our center the frequency of PCO according to our results is approximately 8.5% of the total adolescent population in our area.

These findings show that the teenagers in Thrace are not yet skeptical about the pill’s safety, beneficial effects and correlate the pill use with serious health risks and side effects. Adolescence is a period of rapid physical development triggering the simultaneous secretion of various growth hormones and the teenagers are influential from various information sources to choose consciously for a contraception method adequate to their health condition. In the present study our family center plays a very important role and this is in accordance to literature.

## 5. Conclusions

Ten% of adolescents suffer from chronic diseases and need effective contraception to prevent pregnancy. There are several safe and effective methods of contraception but the choice of the most appropriate one should be personalized due to the complexity of the underlying disease.

For these reasons, proper guidance and monitoring of the implementation of the appropriate contraceptive method of the above teenagers should not be overlooked. The provision of services includes a variety of directions, bearing in mind that the needs of teenagers differ from those of adults.

It should be done in a friendly youth atmosphere in a climate of confidence, taking into account the legal specificities in different countries regarding the age of contraceptive protection. In the provision of services, it is also important to properly assess the particular needs and living conditions of each adolescent. For this reason, international models of advisory guidance have recently been published.

The correct choice of the contraceptive method involves knowing the various methods and the desire for contraception. Full understanding and proper implementation of sexual education facilitates significantly improves the use of contraception.

Typical is the example of Finland combining the application of sex education in the schools with the provision of high-quality health care. These factors have greatly contributed to reduce the rate of unwanted pregnancies and voluntary abortions. On the contrary, the cessation of implementation of the above programs has resulted in contrary trends.

Sexual behavior does not differ significantly in developed countries with respect to the age of 1st sexual intercourse, with an average of 17 years old. Also, 15% of teenage girls had their first sexual intercourse before 15 years of age, with 60% in the 18th year and 80% in the 20th year. However, the incidence of unwanted pregnancies varies, indicating with significant depending on the type of contraceptive method. A combination of a hormonal contraceptive method with male condom is preferred both for contraception and STI protection. Informing adolescent girls with a simple language about all the potential risks and benefits contributing to the proper choice of contraceptive method. The family planning centers are of great importance for organizing various social strategies not limited to formal government programmes. These strategies are based on parameters affecting the application of contraception method such as population socioeconomic status and educational background.

## Figures and Tables

**Table 1:** Epidemiologic data of the study populations.

Epidemiologic Data	No of Women	(%)
Age		
<18 years	99	33.0
≥18 years	201	67.0
Religion		
Muslims	113	37.7
Christians	187	62.3
Way of Living		
Rural	60	20.0
Urban	240	80.0
Occupation		
Student	229	76.3
Employed	39	13.0
Unemployed	32	10.7
Smoking		
No	240	80.0
Yes	60	20.0
Abnormal Bleeding		
No	200	66.7
Yes	100	33.3
Disease in the Past		
No	282	94.0
Yes	18	6.0
Medication		
No	248	82.7
Yes	52	17.3
Acne		
No	221	73.7
Yes	79	26.3
Hirsutismus		
No	226	75.3
Yes	74	24.7
Diabetes Mellitus		
No	299	99.7
Yes	1	0.30
Side Effects	0	0.0
Abort/Pregnacy	0	0.0
Childern		
None	292	97.3
More than one child	8	2.7
Age of Menarche		
Median (25–75% percentile)	12 (11–13)	

**Table 2:** Method of contraception in the study population.

Contraception Method	No of Women	Percentage (%)
Oral contraceptives	168	56.0
Condom	137	45.7
Interrupted coitus	127	42.3
IUD	32	10.7
Spermicides	5	1.7

**Table 3:** (**a**) Different methods of contraception in the study population; (**b**) Source of information regarding contraception.

**(a)**		
**Contraception Method**	**No of Women**	**Percentage (%)**
None	33	11.0
One method	107	35.7
Two methods	124	41.3
Three methods	30	10.0
Four methods	6	2.0
**(b)**		
**Source of Information**	**No of Women**	**Percentage (%)**
Sexual partner	37	12.4
Family center	141	47.0
Family	61	20.3
School	61	20.3

**Table 4:** Use of oral contraceptives, condom and interrupted coitus according to women’s characteristics.

**Epidemiologic Data**	**Women Using OCs (%)**	***p* Value**	**OR (95% CI)**
Age		0.722	
<18 years	54 (54.5)		1
≥18 years	114 (56.7)		1.09 (0.67–1.77)
Religion		**0.015**	
Muslims	53 (46.9)		1
Christians	115 (61.5)		1.81 (1.13–2.90)
Way of Living		**0.012**	
Rural	25 (41.7)		1
Urban	143 (59.6)		2.06 (1.16–3.66)
Occupation		**0.077**	
Student	120 (52.4)		1
Employed	26 (66.7)		1.82 (0.89–3.71)
Unemployed	22 (68.8)		2.00 (0.91–4.41)
	**Women Using CONDOM (%)**	***p* Value**	**OR (95% CI)**
Age		0.331	
<18 years	38 (38.4)		1
≥18 years	89 (44.3)		1.28 (0.78–2.09)
Religion		<0.001	
Muslims	79 (69.9)		6.73 (4.01–11.31)
Christians	48 (25.7)		1
Way of Living		0.683	
Rural	24 (40.0)		1
Urban	103 (42.9)		1.13 (0.63–2.01)
Occupation		0.381	
Student	101 (44.1)		1
Employed	16 (41.0)		0.88 (0.44–1.76)
Unemployed	10 (31.3)		0.58 (0.26–1.27)
	**Women Using C.I. (%)**	***p* Value**	**OR (95% CI)**
Age		0.299	
<18 years	41 (41.4)		1
≥18 years	96 (47.8)		1.29 (0.80–2.10)
Religion		0.566	
Muslims	54 (47.8)		1
Christians	83 (44.4)		0.87 (0.55–1.40)
Way of Living		0.862	
Rural	28 (46.7)		1
Urban	109 (45.4)		0.95 (0.54–1.68)
Occupation		0.927	
Student	106 (46.3)		1
Employed	17 (43.6)		0.90 (0.45–1.78)
Unemployed	14 (43.8)		0.90 (0.43–1.90)

**Table 5:** Use of IUD and spermicides according to women’s characteristics.

**Epidemiologic Data**	**Women Using IUD (%)**	***p* Value**	**OR (95% CI)**
Age		0.824	
<18 years	10 (10.1)		1
≥18 years	20 (10.9)		1.09 (0.50–2.41)
Religion		0.007	
Muslims	19 (16.8)		2.71 (1.28–5.72)
Christians	13 (7.0)		1
Way of Living		0.224	
Rural	9 (15.0)		1
Urban	23 (9.6)		0.60 (0.26–1.38)
Occupation		0.087	
Student	22 (9.6)		0.41 (0.17–1.01)
Employed	8 (20.5)		1
Unemployed	2 (6.3)		0.26 (0.05–1.32)
	**Women Using SPERMICIDE (%)**	***p* Value**	**OR (95% CI)**
Age		0.114	
<18 years	0 (0.0)		
≥18 years	5 (2.5)		n.a.
Religion		0.299	
Muslims	3 (2.7)		1
Christians	2 (1.1)		0.40 (0.07–2.41)
Way of Living		0.260	
Rural	2 (3.3)		1
Urban	3 (1.3)		0.37 (0.06–2.25)
Occupation		0.007	
Student	2 (0.9)		0.11 (0.02–0.66)
Employed	3 (7.7)		1
Unemployed	0 (0.0)		n.a.

**Table 6:** Method of contraception in the participants with ance, hirsutismus and late age of menarche in relation to religion.

Contraception Method	No of Women	Percentage (%)
Muslims		
Oral contraceptives	11	47.8
Condom	15	65.2
Interrupted coitus	13	56.5
IUD	3	13.0
Spermicides	0	0.0
Christians		
Oral contraceptives	34	61.8
Condom	9	16.4
Interrupted coitus	27	49.1
IUD	2	3.6
Spermicides	0	0.0
Total		
Oral contraceptives	45	57.7
Condom	24	30.8
Interrupted coitus	40	51.3
IUD	5	6.4
Spermicides	0	0.0

IUD: Intrauterine device.
